# Concurrent training and intermittent fasting reduce transverse cross-sectional area of cardiomyocytes and body mass in Wistar rats

**DOI:** 10.3389/fphys.2025.1570624

**Published:** 2025-07-30

**Authors:** Henrique Izaias Marcelo, Marina Politi Okoshi, Letícia Estevam Engel, Wanderson da Silva Rosa, Paulo Henrique Aleixo, Guilherme Akio Tamura Ozaki, Everton Alex Carvalho Zanuto, Luiz Carlos Marques Vanderlei, Francis Lopes Pacagnelli, Robson Chacon Castoldi

**Affiliations:** ^1^Department of Physiology and Biophysics, Institute of Biomedical Sciences, University of São Paulo (USP), São Paulo, Brazil; ^2^Department of Physical Education – Presidente Prudente, University of Western São Paulo (UNOESTE), São Paulo, Brazil; ^3^Postgraduate Program in Pathophysiology in Internal Medicine – Botucatu, Paulista State University (UNESP), São Paulo, Brazil; ^4^Postgraduate Program in Animal Science, University of Western São Paulo (UNOESTE), São Paulo, Brazil; ^5^Postgraduate Program in Physical Exercise in HealthPromotion, University North of Paraná (UNOPAR), Londrina, Paraná, Brazil; ^6^Postgraduate Program in Animal Science, Campinas State University (UNICAMP), Campinas, Brazil; ^7^Postgraduate Program in Movement Sciences – PresidentePrudente, Paulista State University (UNESP), São Paulo, Brazil; ^8^Postgraduate Program in Health Sciences, University of Western São Paulo (UNOESTE), Presidente Prudente, Brazil

**Keywords:** cardiomyocyte, exercise, fasting, heart, aerobic, anaerobic

## Abstract

**Methods:**

This study identified the alterations caused by concurrent training and intermittent fasting on the myocardium of rats. In total, 39 adult male Wistar rats were used, divided into four groups: control [C (n = 12)], fasting control [FC (n = 11)], training [T (n = 8)], and fasting training [FT (n = 8)]. The critical load test was performed to evaluate and determine the intensity of effort in aerobic training (AT) (swimming). The resistance training protocol (RT) (anaerobic) consisted of four series of 10 jumps, with overload corresponding to 50% of the body mass of each animal, and 1 min of rest between each series. The concurrent training (CT) was composed of a protocol of AT and RT in the same session. The intermittent fasting period was 12/12 h. At the end of the experiment, the animals were weighed to obtain the Lee index; heart weight was verified, and tissue samples were collected for further histological analysis. After obtaining the data, the Shapiro-Wilk test was performed, followed by a two-way analysis of variance (ANOVA) with Tukey’s post-test for the variables measured at the end of the experiment.

**Results:**

For the variables body mass, feed consumption, and the anaerobic threshold, the FC group showed a greater decrease in the Lee index [(0.28 ± 0.00) p < 0.05)] in relation to the other groups. There were no significant alterations (p > 0.05) in relation to heart weight, fractal dimension, and anaerobic threshold (AnT); finally, both the T and the FT groups presented a significant decrease ([T: (277.3 ± 119.3/FT: 310.5 ± 148.8] p < 0.05) in the transverse cross-sectional area of the cardiomyocytes, when compared to the control groups.

**Conclusion:**

The practice of concurrent training for a period of 4 weeks precipitated a decrease in the transverse cross-sectional area of the cardiomyocytes of Wistar rats. Furthermore, when combined with intermittent fasting, concurrent training not only led to a reduction in the transverse cross-sectional area of the cardiomyocytes but also resulted in a decrease in body mass compared to the isolated concurrent training model.

## 1 Introduction

Several modalities of physical training, performed at moderate to high intensity, are used to promote beneficial alterations in the cardiovascular system, being responsible for greater mitochondrial adaptation, physiological cardiac hypertrophy, and improvements in different cardiac variables (Ghorayeb et al., 2005; Seo et al., 2019). Among these modalities, “concurrent training” (CT) stands out, consisting of performing exercises that stimulate both aerobic and anaerobic demand in a single training session (Hickson, 1980). Studies carried out to evaluate the effects of this training model on the cardiovascular system report greater cardiovascular adaptation when compared to isolated resistance training, and that this adaptation occurs due to the competition between aerobic and anaerobic metabolisms (Coffey and John, 2017). However, if CT is carried out at very high intensities, it can cause negative effects to the practitioner, such as overtraining, which may affect the magnitude of molecular signaling and, consequently, impair protein synthesis, thus generating negative effects at the cellular level (Fyfe, Bishop, Stepto, 2014; Schoenfeld, 2010; Souza et al., 2001).

Some studies have identified that concurrent training is able to alter cardiovascular and muscle health variables to a greater extent when compared to other training modalities. These alterations include a decrease in resting heart rate, systolic and diastolic blood pressure, and an increase in maximal oxygen consumption. (Davis et al., 2008; Häkkinen et al., 2003; Sheikholeslami-Vatani et al., 2015; Takeshima et al., 2004). However, some authors suggest that the excessive fatigue caused by concurrent training (CT) may impair improvements in physical capacities, as CT activates different energy systems simultaneously and compromises the individual’s recovery (Aoki and Gomes, 2005; Bell et al., 2000). It is known that concurrent training uses energy substrates from both anaerobic and aerobic pathways and that fasting promotes less energy availability during its performance, causing the metabolism to use a greater amount of fatty acids as an energy source (Medeiros, et al., 2015; Hill et al., 2012). Intermittent fasting is one method of food restriction, consisting of fasting during the day and feeding only when the sun goes down (Saleh et al., 2005).

Studies indicate that this type of fasting promotes cardiovascular protection against heart disease, decreased heart rate, and protection against muscle injury biomarkers (Saleh et al., 2005; Mrad et al., 2019; Varady et al., 2007). It is also important to acknowledge that high-intensity physical exercise can compromise cardiac function, inducing arrhythmias, heart failure, and even sudden cardiac death. These outcomes may result from enzymatic imbalances, structural damage, and reduced cellular oxygen availability (Junqueira et al., 2016; Ventura-Clapier et al., 2011; Ping et al., 2015). Additionally, low levels of certain nutrients can increase cardiovascular risks; for instance, sodium restriction may trigger several physiological effects associated with cardiac function (Graudal et al., 2014; Paterna et al., 2008; Parrinello et al., 2009). Thus, the authors' hypothesis was that the high intensity of concurrent training (CT), combined with dietary restriction, could lead to changes in cardiac tissue, especially in cardiomyocyte size. This reduction may result from exercise-induced adaptations and altered nutrient availability due to fasting, since cardiomyocytes are responsive to both mechanical and metabolic stimuli (Trager et al., 2023). Therefore, the present study is relevant because it reports the effects of a CT model and intermittent fasting method on the myocardium.

Despite demonstrating different characteristics when compared to humans, such as the circadian cycle, studies involving animals present advantages, for example, the high control of variables and ease of manipulation. Additionally, the effects found can be extrapolated to humans or used to encourage further studies (Quinn, 2015). Thus, the aim of the present study was to verify the possible alterations in the myocardium of rats after 12 sessions of CT associated with intermittent fasting.

## 2 Methods

### 2.1 Ethical approval

The study was developed in compliance with the rules and ethical principles of the Brazilian College of Animal Experimentation (BCAE) and was approved by the Ethics Committee for the Use of Animals (ECUA-5372). The investigators understand the ethical principles under which the journal Frontiers in Physiology – Exercise Physiology operates, and this work complies with the journal’s animal ethics checklist (Grundy, 2015).

### 2.2 Animals

In total, 39 male Wistar rats (*Rattus novergicus albinus*) were used, with a mean age of 150 days and a body mass of 431 ± 2.94 g. The experiment started with 40 animals, however, there was a sample loss due to one animal drowning during the development of the study. The animals were housed in groups of 2 animals per box (polyethylene), with a controlled ambient temperature (22°C ± 2°C) and brightness (light/dark cycle of 12 h), with lights turned on at 10 a.m. and off at 10 p.m.

### 2.3 Division of groups

The animals were divided into four groups:

Control Group [C (n = 12)]: the animals remained loose in their cages, with free access to water and feed (feed for laboratory rats – Primor®).

Fasting Control [FC (n = 11)]: the animals remained loose in their cages, with free access to water, however, feeding was controlled to a fasting period of 12 h for 12 h of free access to feed (Ferreira Da Silva et al., 2011).

Training [T (n = 8)]: This training model was composed of two different exercise sessions, which require different energy sources during execution, one being predominantly aerobic (swimming) and the other predominantly anaerobic (jumping). Access to water and feed was *ad libitum*.

Fasting Training [FT (n = 8)]: This group was identical to the T group except that the animals fasted, under the same model that was applied to the FC group (12 × 12 h).

### 2.4 Concurrent training

Prior to beginning the training protocols, the rats were submitted to a period of adaptation to the liquid environment and equipment (10–20 min/day, 3 days a week, for 1 week, with progressively increasing overload and duration) (Manchado et al., 2006). An adaptation period reduces the stress produced by the liquid medium and by the physiological alterations resulting from physical training (Chimin et al., 2009).

The aerobic training protocol was performed first, followed by the resistance training protocol. The protocols were performed sequentially, with a pause only to change the training area. The training was carried out three times a week for 1 month, totaling twelve training sessions. The aerobic training protocol consisted of three weekly sessions on non-consecutive days, comprising 30 min of swimming exercise, with an intensity of 80% of the anaerobic threshold (AnT), stipulated from the critical load test (Manchado et al., 2006). For the swimming exercise, a tank was used, containing cylindrical polyvinyl chloride (PVC) tubes, 25 cm in diameter and 100 cm in height, with water at a depth of 70 cm, and a controlled water temperature (Ozaki et al., 2014).

**FIGURE 1:**
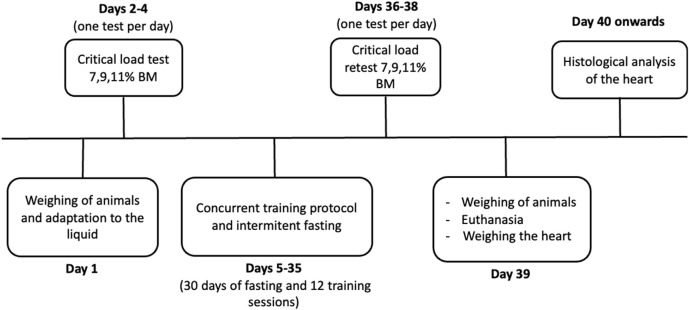
Experimental design. BM = Body Mass.

For the resistance training protocol, four series of 10 jumps were used, with overload, adjusted weekly, corresponding to 50% of the body mass of each animal. A polyvinyl chloride (PVC) tube, 25 cm in diameter, 80 cm in height, and 38 cm deep was used. The overload was carried in a waistcoat made from elastic, with a Velcro closure, attached to the chest area, specially designed for this type of exercise (Hill, 1993) (Figure 1).

### 2.5 Critical load test

The critical workload (CWL) and anaerobic capacity (CTA) were obtained by inducing swimming exercise with three different stimuli. Three loads were selected for each animal, corresponding to 7, 9, and 11% of body mass, so that the animals performed all efforts (Hill, 1993).

The exercises were chosen with the intention of the animals entering exhaustion after between 2 and 10 min (Hill, 1993). In this way, we recorded the time limit (Tlim) to perform the exercise at each of the loads. The animals rested for 24 h after each stimulus (Marangon et al., 2002; Chimin et al., 2009; Castoldi et al., 2013). The values established for the two variables were obtained using the formula: Critical Load = CWL+ (CTAx1/Tlim).

After performing this procedure, the aerobic capacity of each animal could be determined, which was necessary to identify the intensity of effort in the swimming exercise.

### 2.6 Food consumption

Food consumption was recorded at each change of feed and water to establish parameters between the groups, calculated by the ratio between the animal’s body mass (BManimal) and food consumption, both in grams (g). Consumption was calculated by the content of feed offered (FO) and subtracted from the surplus (S), using the formula [BManimal/FO-S] (De Luca et al., 1987).

### 2.7 Intermittent fasting protocol

Intermittent fasting was performed with a 12/12 protocol, that is, the animals performed 12 h of fasting with 12 h of feeding on every day of the week (Ferreira Da Silva et al., 2011). The fasting period started at 9 p.m. and ended the next day at 9 a.m. The training took place 1 h before the beginning of the feeding period, so the animals performed the concurrent training while still fasting. Water consumption was *ad libitum*, 24 h a day, for all animals.

### 2.8 Lee Index

**FIGURE 2:**
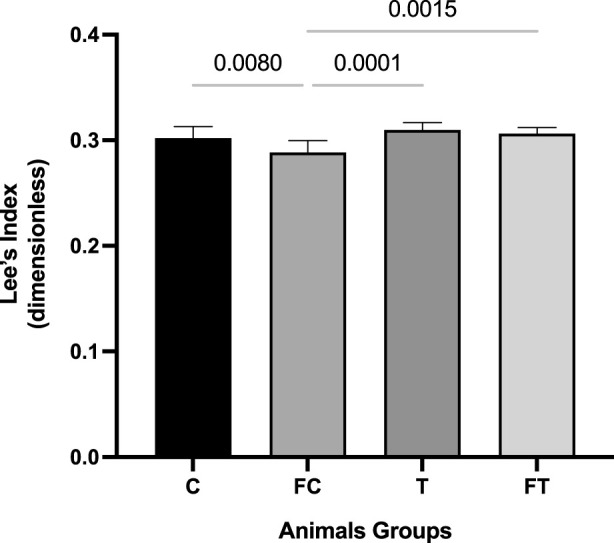
Comparison of the Lee Index between groups after intervention. C (12) = Control; FC (11) = Fasting control; T (8) = Training; FT (8) = Fasting training. Statistically significant difference of the *two-way ANOVA* test with *Tukey’s* post-test with 5% significance (p < 0.05).

The Lee index was calculated for all animals, using the ratio between the cube root of body mass (g) and the naso-anal (muzzle-coccyx) length in centimeters, as described by Novelli and colleagues (2007). Although this method provides a practical and non-invasive approach to estimate body adiposity in rodents, it represents an indirect measure and does not allow precise quantification of fat mass. Therefore, interpretation of this index should be made with caution, and future studies are encouraged to include direct assessments of body composition, such as dissection-based fat pad analysis or imaging techniques (Figure 2).

### 2.9 Anesthetic protocols

The animals were euthanized 48 h after the final training session to avoid the influence of acute exercise-induced effects on the analyses and outcomes. Anesthesia was induced using an intraperitoneal injection of ketamine hydrochloride (40 mg/kg) and xylazine hydrochloride (10 mg/kg). Subsequently, the animals were euthanized by exsanguination via cardiac puncture (Castoldi et al., 2013).

### 2.10 Surgical procedures and weighing the heart

After euthanasia, the heart of each animal was surgically removed, and the ventricles and atria were removed and weighed separately on a precision scale, Kern & Sohn® (model PNJ 600-3M, manufactured in Balingen-Frommern, GER), and an analytical scale of the brand Marte Científica® (model AD500, manufactured in São Paulo, BRA) (Pinheiro et al., 2018), before being stored in a formaldehyde solution (10%) for histological processing (Gimenes et al., 2015).

### 2.11 Histological processing of the cardiac muscle

Samples of the left ventricle were fixed in a 10% buffered formaldehyde solution for a period of 48 h, after which the tissue was embedded in paraffin blocks, for later preparation of the histological slides. After fixation, the hearts were sectioned transversely immediately below the coronary sulcus, and longitudinally, to obtain only the left ventricles. Next, the hearts were washed, dehydrated, and embedded in paraffin (Noh et al., 2015). The blocks containing the fragments of the cardiac tissue were cut in a rotating microtome using the semi-serial method, with thicknesses of 5 µm.

The sections were subjected to Hematoxylin-Eosin (HE) staining for morphological-histomorphometric analysis (Ozaki et al., 2015).

### 2.12 Histological analysis

For the histomorphological analysis, to quantitatively evaluate the tissues, images of the slides stained with HE were obtained using the Nikon® eclipse 50i (manufactured in New York, United States) optical microscope attached to an Infinity 1 camera (manufactured in California, United States).

For the histomorphometric analysis, to qualitatively evaluate the tissues, the sectional area of cardiomyocytes was measured using NIS-Elements, advanced image acquisition, analysis and processing software developed by Nikon®. For this, images were used with a ×40 magnification, and 50 cardiomyocytes from each animal were measured. For the analysis of the left ventricle, a Leica® optical microscope (DM500 model, manufactured in Illinois, United States) was used, with Leica Application Suite software version 4.2.0. The measurement was performed through the left ventricle contour, where, at the end of the contour, the software recorded the final size of the contoured line in a micrometer (Junqueira et al., 2016).

### 2.13 Data analysis

After obtaining the data, the Shapiro-Wilk normality test was performed and the comparison between groups was carried out through the analysis of variances (two-way ANOVA). This procedure was performed for all variables obtained at the end of the experiment. In addition, the two-way ANOVA test with repeated measures was utilized and Tukey’s post-test for body mass, feed consumption, and the anaerobic threshold. All procedures adopted a significance value of 5% (p < 0.05). The calculations were performed using the statistical package (GraphPad Prism v.10.0. for Mac®).

## 3 Results

### 3.1 Body mass

**FIGURE 3:**
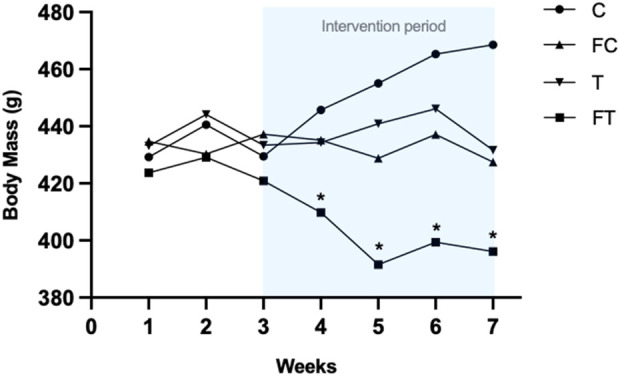
Body mass of groups during the evaluations. C (12) = Control; FC (11) = Fasting control; T (8) = Training; FT (8) = Fasting training. Significant difference between FT and C, FC, and T. * = statistically significant difference vs. control group.

The FT group presented decreases in body mass throughout the experiment, leading to a statistically significant decrease in mass (g) in relation to groups C (p = 0.0001), FC (p = 0.007), and T (p = 0.001). After statistical analysis, the final body mass means of all groups throughout the intervention were as follows: C (468.5 ± 20.7); FC (427.4 ± 33.5); T (431.6 ± 26.7); and FT (396.1 ± 26.3) (Figure 3).

### 3.2 Lee index

The FC group showed a significant decrease for the Lee’s Index variable when compared to the other groups. The means and standard deviations of the groups were: C: 0.30 ± 0.01; FC: 0.28 ± 0.00; T: 0.30 ± 0.00; and FT: 0.30 ± 0.00.

### 3.3 Feed consumption

**FIGURE 4:**
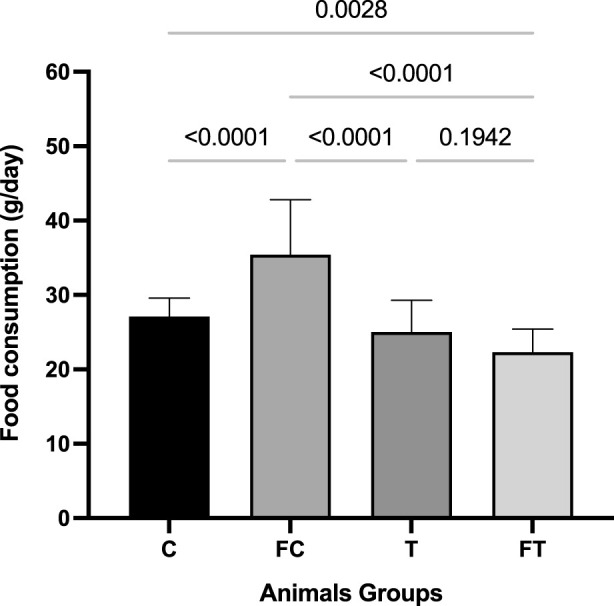
Comparison of Feed Consumption between groups after the intervention. C (12) = Control; FC (11) = Fasting control; T (8) = Training; FT (8) = Fasting training. Statistically significant difference by *two-way ANOVA* test with repeated measures and *Tukey’s* post-test (*p* < 0.05).

A significant increase in feed consumption was verified in the FC group when compared with the other groups. The groups demonstrated the following respective means: (C = 27.12 ± 0.49); (FC = 35.41 ± 1.48); (T = 26.20 ± 1.45); (FT = 22.31 ± 0.62). After the statistical analysis of feed consumption, significant differences were noted between group FC and groups C (p = <0.0001) and FT (p = 0.0015); and between group FC (p = 0.001), and groups T and FT (p = 0.001). This result can to be see in Figure 4.

### 3.4 Anaerobic threshold

**FIGURE 5:**
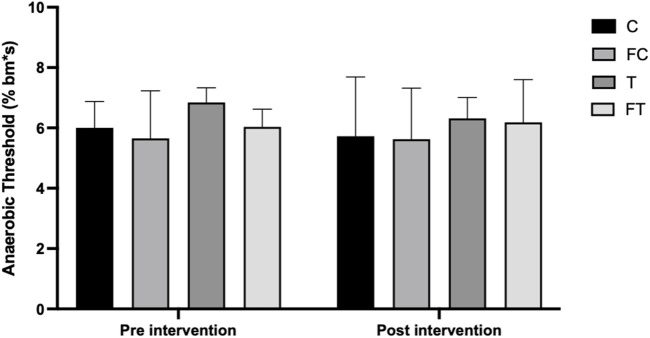
Pre and post anaerobic threshold of the groups. C (12) = Control; FC (11) = Fasting control; T (8) = Training; FT (8) = Fasting training. Legend: BM = Body Mass; s = seconds.

The pre-intervention Anaerobic Threshold means, expressed in % body mass × seconds (%bm*s), were as follows: (C = 6.00 ± 0.22); (FC = 5.65 ± 0.52); (T = 6.84 ± 0.17); (FT = 6.03 ± 0.20), and after 4 weeks of intervention, thpe means were: (C = 6.11 ± 0.37); (FC = 5.62 ± 0.83); (T = 6.64 ± 0.15); (FT = 6.47 ± 0.26). The results of the Anaerobic Threshold before and after the intervention did not show significant alterations after performing the statistical analysis (Figure 5).

### 3.5 Heart weight

The results found related to the weight of the heart were not significant after performing the statistical test (Table 1).

**TABLE 1:** Results, with mean, standard deviation and significance of heart weight.

Variables	C	FC	T	FT	P
Δ Body Mass	+39,4	−7	−13	−32	-
THW (g)	1.25 ± 0.11	1.23 ± 0.19	1.22 ± 0.06	1.17 ± 0.07	0.492
(% Body Mass)	0.28 ± 0.019	0.24 ± 0.012	0.30 ± 0.025	0.29 ± 0.026	0.131
RV (g)	0.24 ± 0.07	0.21 ± 0.19	0.20 ± 0.03	0.20 ± 0.04	1.000
(% Body Mass)	0.053 ± 0.005	0.052 ± 0.000	0.056 ± 0.011	0.055 ± 0.017	0.074
LV (g)	0.94 ± 0.08	0.95 ± 0.18	0.93 ± 0.05	0.88 ± 0.07	0.672
(% Body Mass)	0.21 ± 0.013	0.17 ± 0.017	0.22 ± 0.021	0.22 ± 0.013	0.961
R/L Atrium (g)	0.07 ± 0.01	0.06 ± 0.01	0.08 ± 0.02	0.07 ± 0.03	1.000
(% Body Mass)	0.018 ± 0.002	0.018 ± 0.005	0.020 ± 0.005	0.015 ± 0.004	0.808

Legend: C (12), Control; FC (11), Fasting control; T (8), Training; FT (8), Fasting training. THW, total heart weight; RV, right ventricle; LV, left ventricle; R/L Atrium R/L, Right/Left Atrium. % Body Mass: Percentage value in relation to total body mass.

### 3.6 Transverse cross-sectional area of cardiomyocytes

**FIGURE 6:**
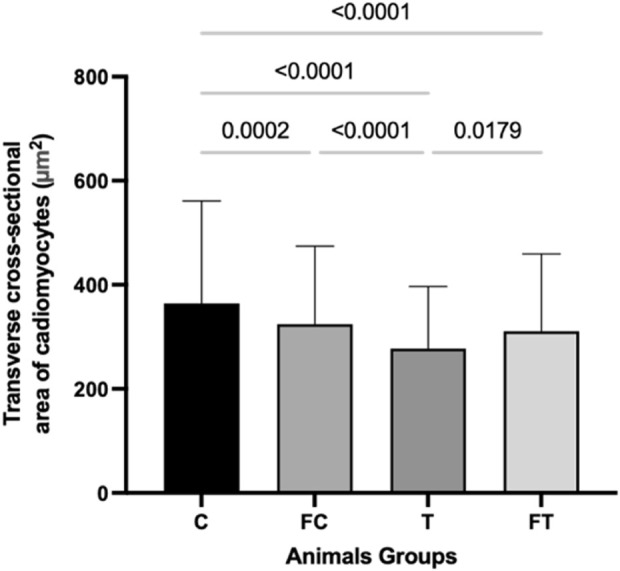
Comparison of transverse cross-sectional area of cardiomyocytes (µm^2^) between groups. C (12) = Control; FC (11) = Fasting control; T (8) = Training; FT (8) = Fasting training. Statistically significant difference of the *two-way ANOVA* test with *Tukey’s* post-test with 5% significance (p < 0.05).

After observing the area of the cardiomyocytes of the different groups, the following medians and standard derivations were obtained: C (364.1 ± 197.2); FC (324.2 ± 150.1); T (277.3 ± 119.3); and FT (310.5 ± 148.8). A statistically significant decrease was found in the T group in relation to groups FT (p = 0.0179), FC (p = 0.0001) and C (p = <0.0001). In addition, there was also a significant decrease in the transverse cross-sectional area of cardiomyocytes in the FT group compared to group C (p = <0.001) (Figure 6).

### 3.7 Left ventricular lumen diameter

**FIGURE 7:**
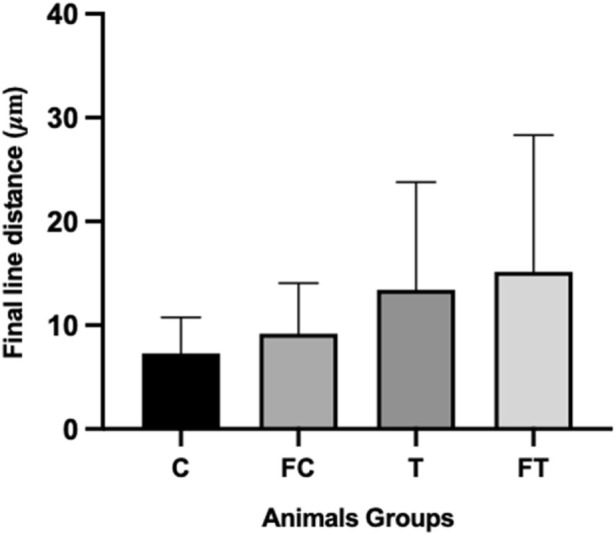
Final distance of left ventricle lumen contour of the different sample groups. C (12) = Control; FC (11) = Fasting control; T (8) = Training; FT (8) = Fasting training.

Post statistical analysis, the following results were found: C (27 ± 1.04); FC (9.18 ± 1.4); T (13.92 ± 3.91); and FT (15.14 ± 4.97). Although the trained groups presented higher values, no significant differences were verified in left ventricle lumen size and heart size (Figure 7).

**FIGURE 8:**
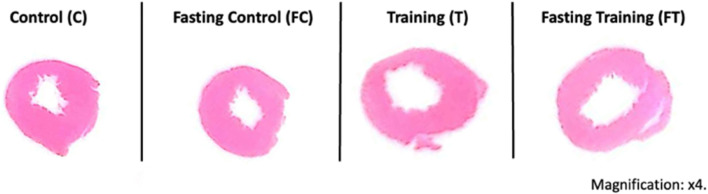
Histomorphology of cross-sections of the striated cardiac muscles of the different sample groups. C (12) = Control; FC (11) = Fasting control; T (8) = Training; FT (8) = Fasting training.

When performing a qualitative comparison of the cardiac histomorphology of the different sample groups, a visually apparent increase in the left ventricular lumen was observed in the trained groups (T and FT), compared to control groups (C and FC), although no statistical analysis was performed (Figure 8).

## 4 Discussion

The main finding of this study was that concurrent training promoted cardiac remodeling, characterized by a decrease in the cardiomyocyte diameter, when compared to the control group.

Interestingly, although increased intraventricular pressure and wall stress are generally expected to promote cardiomyocyte hypertrophy, since larger fibers contain more contractile elements arranged in parallel, allowing for greater force production (Rassier, 2017; Nakamura and Sadoshima, 2018), the results of the present study demonstrated a reduction in cardiomyocyte size. This apparent contradiction suggests that alternative adaptive mechanisms may be involved, particularly in the context of aerobic exercise. A plausible hypothesis is that the decrease in cardiomyocyte diameter represents physiological remodeling aimed at enhancing oxygen diffusion. In this regard, it is possible that smaller cells reduce the distance between capillaries and mitochondria, facilitating the oxygen transport needed to meet the increased metabolic demand during exercise (Kayar and Weiss, 1992; Poole and Mathieu-Costello, 1996). This interpretation aligns with the aerobic nature of the training protocol and may reflect a beneficial myocardial adaptation to optimize efficiency under conditions of elevated cardiac output, since in skeletal muscle, smaller fibers and greater capillary density reduce the diffusion distance, improving oxygen transport during exercise (Hellsten and Gliemann, 2024). However, further studies that evaluate the relationship between cardiomyocyte diameter, mitochondrial distribution, capillary distance, and oxygen delivery are needed, especially in the context of aerobic training, to validate this hypothesis.

When comparing the transverse cross-sectional area of the cardiomyocytes, an important sum of sides that reveals the outline of each cell, between the T and FT groups, a decrease was noted in the T group. This can be justified by the anti-apoptotic and anti-inflammatory protection that fasting offers cardiomyocytes (Ahmet et al., 2005). However, some studies have identified that high intensity exercise can prevent skeletal muscle atrophy and that fasting can cause atrophy in this type of muscle (Belavý et al., 2017; Masiero et al., 2009).

On the other hand, with regard to cardiac muscle tissue, it was observed that high-intensity training is related to left ventricular morphological alterations (Dores et al., 2018). In addition, studies show that insulin can attenuate the mechanism of atrophy and autophagy of genes related to cardiomyocytes, through metabolic pathways involving AKT protein phosphorylation and FOXO3a inactivation (Paula-Gomes et al., 2013). Thus, there is a hypothesis that training combined with fasting may have somehow influenced body response to insulin secretion, as there was a greater decrease in cardiomyocytes in the group that performed the isolated training. Studies involving this theme and that evaluate hormonal concentrations may contribute to the results of the current research. Another hypothesis is that a physiological cardiac adaptation to exercise may have occurred, leading to eccentric hypertrophy of the cardiomyocytes. In this case, there would be an increase in sarcomeres in relation to cell length without compromising cardiac functionality, which is expected (Nakamura and Sadoshima, 2018).

In line with the present study, other research has also shown that athletes who engage in high-intensity physical exercise may exhibit an increase in left ventricular lumen, as a physiological adaptation to the increased cardiac demand induced by exercise (Lang et al., 2006; Galderisi et al., 2015; D'Andrea et al., 2017). This type of cardiac remodeling is commonly observed in trained individuals and may represent a functional response to the volume and intensity of training. However, in some cases, this structural enlargement may be associated with greater ventricular wall stress during systole, and even the development of arrhythmias. Thus, it is hypothesized that concurrent training, due to its intensity, may induce structural adaptations in the heart, such as an increase in the left ventricular cavity, possibly accompanied by adjustments in cardiomyocyte size.

Although this variable is an indirect analysis of body adiposity, there was a significant decrease in values related to the Lee index in the T group in relation to the other groups; a decrease in the transverse cross-sectional area of cardiomyocytes of animals in the FT group, when compared with C and FC; and no significant alterations in anaerobic threshold values and heart weight. However, expansion of the cardiac chamber was observed in the trained animals (groups T and FT). This may be justified by the fact that the myocardium, in order to adapt to exercise and maintain hemodynamic homeostasis, adapts morphologically through ventricular dilation, especially the left ventricle.

In the present study, it was evidenced that the Lee Index of the FC group and of the trained groups decreased in relation to the control group. Thus, the present study corroborates the study by Machado and colleagues (2014), in which a decrease in the Lee index of animals practicing CT was found (Machado et al., 2014). This decrease may have occurred because physical training is able to decrease body fat and physical inactivity causes a decrease in muscle mass (Kang et al., 2009; Janssen et al., 2002). The Lee index was significantly lower in the FT group when compared to the T group. This can be explained by the significant decrease in body mass in the FT group, because the greater the fat loss, the lower the Lee index (Souza et al., 2001).

Unlike the Lee index, there were no alterations in the anaerobic threshold, or heart weight values between groups. A study involving different training models showed that training with continuous load promoted an increase in heart weight in relation to the control group and the group that performed the training with progressive loads (Rocha et al., 2008). Thus, there is a hypothesis that the volume of this training model is more suitable for this cardiac adaptation. On the other hand, high-intensity interval training, over a 6-week period, did not significantly alter heart variables or weight (Songstad et al., 2007).

Regarding the anaerobic threshold (AnT) evaluations, although the groups did not present a statistically significant difference between the pre and post intervention moments, a slight decrease in the AnT of the animals in the training group was noted compared to the control group. This fact may have occurred due to the increase in body fat in animals in the C and FC groups, which favors buoyancy, while muscle hypertrophy of the lower limbs, generated by training, ends up impairing the buoyancy of animals in the T group (Castoldi et al., 2016).

Regarding food consumption, both the animals that performed the concurrent training, as well as those that performed fasting coupled with training, presented significantly reduced feed intake, in relation to the control group. These results are in favor of the findings of Machado and colleagues (2014), who identified lower food consumption of animals that practiced concurrent training for 4 weeks, compared to the control group. It is noteworthy that, in the present study, the fasting control group presented a significant increase in food consumption compared to the other groups, which can be explained by the decrease that fasting causes on nocturnal leptin levels (Alzoghaibi et al., 2014).

It is important to acknowledge that the present study has limitations. Notably, it was not possible to perform functional cardiac assessments, such as echocardiography, hemodynamic analysis, or direct evaluation of myocardial contractility. The study also lacked molecular analyses that could help distinguish adaptive from maladaptive remodeling processes. These limitations restrict more comprehensive understanding of the physiological relevance of the observed morphological alterations. Despite these constraints, this study contributes to the literature by exploring the effects of concurrent training and intermittent fasting on the cardiac tissue of Wistar rats. Future studies should aim to include functional and molecular assessments, investigate different training modalities, and incorporate additional analyses, such as citrate synthase activity or protein expression, in order to better elucidate the underlying mechanisms and validate the structural findings reported herein.

In summary, it is concluded that the practice of concurrent training for a period of 4 weeks induced a decrease in the transverse cross-sectional area of cardiomyocytes in Wistar rats. Furthermore, when combined with intermittent fasting, concurrent training not only led to a reduction in transverse cross-sectional area of the cardiomyocytes, but also resulted in a decrease in body mass compared to the isolated concurrent training model.

## Data Availability

The raw data supporting the conclusions of this article will be made available by the authors, without undue reservation.
