# Supplementary material for: Concurrent training and intermittent fasting reduce transverse cross-sectional area of cardiomyocytes and body mass in Wistar rats
Source: Front Physiol. 2025 Jul 30;16:1570624. doi: 10.3389/fphys.2025.1570624 (PMC12343495; doi:10.3389/fphys.2025.1570624)
Supplement: Supplementary file 1 [file DataSheet1.pdf]

Statistical Summary Document

Manuscript Title: Concurrent Training and Intermittent Fasting Reduce Transversal Sectional Area of Cardiomyocytes and Body Mass in Wistar Rats.

Authors: Henrique Izaias Marcelo, Marina Politi Okoshi, Letícia Estevam Engel, Wanderson da Silva Rosa, Paulo Henrique Aleixo, Guilherme Akio Tamura Ozaki, Everton Alex Carvalho Zanuto, Luiz Carlos Marques Vanderlei, Francis Lopes Pacagnelli, Robson Chacon Castoldi.

Animal model used, if applicable: Adult male Wistar rats.

Underlying hypothesis: The hypothesis was that the high intensity of Concurrent Training (TC), added to dietary restriction, could cause tissue alterations in the heart.

Definitions of 'n':

Question 1: Body Mass of groups. n = 39
Question 2: Lee Index of groups. n =39
Question 3: Food consumption of groups. n = 39
Question 4: Anaerobic threshold of groups. n = 39
Question 5: Total heart weight (THW). n = 39
Question 6: Right Ventricle weight. n = 39
Question 7: Left Ventricle weight. n = 39
Question 8: Atrium right and left weight. n = 39
Question 9: Fractal dimension. n = 39.
Question 10: Transverse sectional area of cardiomyocytes.
n = 39 Question 11: Final distance of left ventricle lumen.
n = 39

Statistical summary table:

Experimental question number*	Finding/ conclusion	Experimental location/ variable e.g. muscle, neocortex or genotype	Mean value (or other summary statistic)	SD	n val.	P**	Units	Statistical test	Any other variable e.g. subjects' age or sex	Figure/ table in which data are presented	Comments e.g. observation
1. Will be a	The FT group	Control			39	-	12		None.	Figure 2	Body mass (g).

Statistical Summary Document

change in body mass?	demonstrated decrease in body mass.	group (C)	468.5	20.7							
		Fasting control group (FC)	427.4	33.5	39	FC vs C **0.0109**	11	Two-way ANOVA test with repeated measures and Bonferron i´s post- test		Figure 2	
		Training group (T)	431.6	26.7	39	T vs FT **<0.0001**	8			Figure 2	
		Fasting Training group (FT)	396.1	26.3	39	FT vs C **<0.0001** FT vs FC **<0.0001**	8			Figure 2	
2. Will be a change in Lee Index?	The FC group demonstrated decrease in Lee Index when compared to other groups	Control group (C)	0.30	0.01	39	-	12	Two-way ANOVA test with Tukey's post-test.	None.	Figure 3	Lee Index = ratio between the cube root of body weight in grams (g) divided by muzzle-coccyx length (cm).
		Fasting control group (FC)	0.28	0.01	39	FC vs C **0.008**	11			Figure 3	
		Training group (T)	0.30	0.007	39	T vs FC **0.0001**	8			Figure 3	
		Fasting Training group (FT)	0.30	0.005	39	FT vs FC **0.0015**	8			Figure 3	

Statistical Summary Document

3. Which group will have highest food? consumption ?	The FC group demonstrated greater food consumption when compared to other groups.	Control group (C)	27.12		2.47		39	-	12	ANOVA test with repeated measures and Tukey's post-test.	None.	Figure 4	None.
		Fasting control group (FC)	35.41		7.40		39	FC vs C **0.008**	11			Figure 4	
		Training group (T)	26.20		7.25		39	T vs FC **0.0001**	8			Figure 4	
		Fasting Training group (FT)	22.31		3.10		39	FT vs FC **0.0015**	8			Figure 4	
			Pre	Post	Pre	Post							
4. There were changes in the in the anaerobic threshold?	Not significant difference was seen between groups.	Control group (C)	5.92	5.90	0.88	2.15	39	-	12	Two way ANOVA test with repeated measures and Bonferroni´s post-test.	None.	Figure 5	None.
		Fasting control group (FC)	5.56	5.52	1.66	1.97	39	FC vs T 0.11	11			Figure 5	
		Training group (T)	6.84	6.31	0.48	0.69	39	T vs FT 0.71	8			Figure 5	
		Fasting Training group (FT)	6.03	6.18	0.58	1.42	39	FC vs FT 0.58	8			Figure 5	
5. There were difference in the total heart weight	There were no significant changes in THW.	Control group (C)	1.25		0.11		39	-	12	Two-way ANOVA test with Tukey's	None.	Table 1	None.
		Fasting control	1.23		0.19		39	FC vs T 0.68	11			Table 1	

(THW)?		group (FC)						post-test.			
		Training group (T)	1.22	0.06	39	T vs FT 0.82	8			Table 1	
		Fasting Training group (FT)	1.17	0.07	39	FC vs FT 0.68	8			Table 1	
6. There were difference in Right Ventricle weight?	There were no significant changes in Right Ventricle weight.	Control group (C)	0.24	0.07	39	-	12	Two-way ANOVA test with Tukey's post-test.	None.	Table 1	None.
		Fasting control group (FC)	0.21	0.01	39	FC vs T 0.99	11			Table 1	
		Training group (T)	0.20	0.03	39	T vs FT 1.0	8			Table 1	
		Fasting Training group (FT)	0.20	0.04	39	FC vs FT 0.99	8			Table 1	
7. There were difference in Left Ventricle weight?	There were no significant changes in Left Ventricle weight.	Control group (C)	0.94	0.08	39	-	12	Two-way ANOVA test with Tukey's post-test.	None.	Table 1	None.
		Fasting control group (FC)	0.95	0.18	39	FC vs T 0.97	11			Table 1	
		Training group (T)	0.93	0.05	39	T vs FT 0.81	8			Table 1	
		Fasting Training	0.88	0.07	39	FC vs FT	8			Table 1	

Statistical Summary Document

		group (FT)				0.51					
7. There were difference in atrium right and left weight?	There were no significant changes in atrium right and left weight.	Control group (C)	0.07	0.01	39	-	12	Two-way ANOVA test with Tukey's post-test.	None.	Table 1	None.
		Fasting control group (FC)	0.06	0.01	39	FC vs T 0.62	11			Table 1	
		Training group (T)	0.08	0.02	39	T vs FT 0.93	8			Table 1	
		Fasting Training group (FT)	0.07	0.03	39	FC vs FT 0.62	8			Table 1	
9. There were difference in Transverse cross-sectional area of cardiomyocytes?	The T group showed decrease of Transverse cross-sectional area of cardiomyocytes	Control group (C)	364.1	197.2	39	-	12	Two-way ANOVA test with Tukey's	None.	Figure 6	None.
		Fasting control	324.2	150.1	39	FC vs T <**0.0001**	11			Figure 6	

Statistical Summary Document

es?	tes when compared to others groups.	group (FC)						post-test.			
		Training group (T)	277.3	119.3	39	T vs FT **0,0179** T vs C **<0.0001**	8			Figure 6	
		Fasting Training group (FT)	310.5	148.8	39	FC vs FT 0,22 FT vs C **<0.0001**	8			Figure 6	
10. There were difference in final distance of left ventricle lumen?	Although the groups T and FT showed increased lumen, no significant changes were noted.	Control group (C)	7.27	3.46	39	-	12	Two-way ANOVA test with Tukey's post-test.	None.	Figure 7	The left ventricle lumen can be seen in figure 9.
		Fasting control group (FC)	9.18	4.87	39	FC vs T 0.42	11			Figure 7	
		Training group (T)	13.42	10.35	39	T vs FT 0.97	8			Figure 7	
		Fasting Training group (FT)	15.14	13.15	39	FC vs FT 0.42	8			Figure 7	

*You may use multiple lines for the same question to indicate multiple comparisons

**Authors may wish to make the text bold where p is considered significant against a stated confidence limit.
